# A Novel Nano-Hydroxyapatite Agarose-Based Hydrogel for Biomimetic Remineralization of Demineralized Human Enamel: An in-vitro Study

**DOI:** 10.2147/CCIDE.S478045

**Published:** 2024-11-02

**Authors:** Sara El Moshy, Israa Ahmed Radwan, Manal Matoug-Elwerfelli, Ahmed Abdou, Marwa M S Abbass

**Affiliations:** 1 Oral Biology Department, Faculty of Dentistry, Cairo University, Cairo, Egypt; 2 Pre-clinical Oral Sciences Department, College of Dental Medicine, QU Health, Qatar University, Doha, Qatar; 3 Department of Restorative Dentistry, Faculty of Dentistry, Universiti Malaya, Kuala Lumpur, Malaysia

**Keywords:** agarose hydrogel, biomimetic, enamel matrix derivatives, nano-hydroxy apatite, remineralization

## Abstract

**Purpose:**

This study aims to investigate the biomimetic effect of agarose hydrogel loaded with enamel matrix derivative (EMD-agarose) alone or in combination with nano-hydroxyapatite (n-HA-EMD-agarose) on the remineralization of human demineralized enamel.

**Methods:**

Extracted human mandibular third molars were sectioned into 54 buccal and lingual halves. Acid-resistant nail varnish was applied to each half, except for two enamel windows. Enamel surface microhardness, energy-dispersive X-ray spectroscopy (EDX), and scanning electron microscopy (SEM) analyses were conducted to evaluate enamel surfaces at baseline, following demineralization with 37% phosphoric acid, and after each hydrogel application and remineralization for two, four, and six days. Remineralization was performed using a phosphate solution at 37°C.

**Results:**

At day 6 following remineralization, a statistically significant higher mean microhardness was recorded in n-HA-EMD-agarose hydrogel (260.87 ± 3.52) as compared to EMD-agarose hydrogel (244.63 ± 2.76) (p = 0.027). Similarly, n-HA-EMD-agarose hydrogel showed a higher mean calcium (46.31 ± 2.78), phosphorous (24.92 ± 0.826), and fluoride (0.909 ± 0.053) weight percentage compared to EMD-agarose hydrogel calcium (19.64 ± 1.092), phosphorous (19.64 ± 1.092), and fluoride (0.7033 ± 0.0624) weight percentage (p < 0.05). Further, SEM analysis revealed a substantial deposition of n-HA following the application of the n-HA-EMD-agarose hydrogel, whereas the EMD-agarose exhibited a relatively smooth enamel surface with less visible enamel rods due to mineral deposition.

**Conclusion:**

The combined n-HA-EMD-agarose hydrogel demonstrated improved surface microhardness of the remineralized enamel and enhanced mineral content deposition, indicating its potential as a biomimetic approach for dental enamel repair.

## Introduction

Dental caries is a prevalent chronic disease affecting individuals globally,^1^ with recent statistics indicating that approximately 3.5 billion people are impacted by oral diseases, primarily dental caries.^2^ Structurally, dental enamel is a highly mineralized non-living tissue composed of an inorganic matrix (96 wt.%, mainly hydroxyapatite), organic matrix (proteins and lipids) and water.^3^ Enamel is particularly susceptible to demineralization due to adverse environmental factors such as acid attacks, bacterial by-products, and tooth surface wear.^4^

The balance between remineralization and demineralization is critical; an imbalance can lead to the loss of tooth minerals and the progression to cavitated lesions.^5^ Early-stage carious lesions, non-cavitated, are often clinically identifiable by changes in enamel surface color and glossiness due to prolonged demineralization.^6^ Minimally invasive dentistry focuses on managing initial non-cavitated lesions to prevent further caries progression and to conserve natural tooth structure.^5^ A fundamental principle of this approach is the biomimetic replacement of lost or damaged tooth structure with materials that closely imitate living tissues, restoring natural biological functions such as aesthetics, strength, and function.^7^

Biomimetic mineralization strategies have gained attention for their potential to design templates of organic molecules that facilitate the nucleation and growth of mineralized crystals, as well as mineral ion transport.^8^ Various cell-free strategies have been proposed for repairing enamel structural defects and regenerating the enamel microstructure including, tricalcium phosphate based, casein phosphopeptide-stabilized amorphous calcium phosphate complexes, self-assembling peptide, nano-hydroxyapatite (n-HA), ozone, fluoride, sealant and strontium-containing fluorophosphate glasses-based approaches.^4^,^9^,^10^ In addition to the above named strategies, agarose has garnered interest due to its versatile biological activity, excellent tissue compatibility, low toxicity, and cost-effective production.^11^,^12^ The natural polymer agarose is made up of repeating units of D-galactose and 3,6-anhydro L-galactose. Agarose functions as an organic matrix template for biomimetic mineralization when loaded with calcium and phosphate ions. This results in the formation of precursors known as agarose fiber-nanoscale-amorphous calcium phosphate complex.^4^,^13^,^14^ Mineral precursors are held in reserve by the hydrogen present in agarose, and the limited space in the gel network aids in keeping the size of these complexes consistent and under control.^15^,^16^ Following this, on the calcified collagen fibrils, HA crystals nucleate and grow, aligning their c-axis perpendicular to the dentin surface and eventually forming a densely packed layer of HA similar to enamel.^13^

Additionally, Emdogain (Straumann, Basel, Switzerland), a commercially available enamel matrix derivative (EMD), plays a significant role in enamel crystal orientation, maturation, and biomineralization during tooth formation.^11^,^17^ Amelogenin solution used in-vitro in treating demineralized enamel restored the hardness and the shear bond strength of the samples to levels comparable to those of sound enamel.^18^ EMD also proved to promote the remineralization of enamel in-vitro.^11^,^19^,^20^ Clinical interest has also shifted towards n-HA as an attracted material with antibacterial efficacy and bioactive properties.^21^ In dentistry, n-HA has been applied in various forms and delivery systems for the biomimetic repair of damaged dental enamel,^22^,^23^ leveraging its chemical structure similarity to dental enamel in which the basic enamel building blocks comprise 20–40 nm n-HA.^24^ Moreover, recently, eggshell derived n-HA and n-HA-based composites proved their efficacy in biomimetic dentin remineralization.^25–27^ Yet, numerous variables can affect n-HA’s solubility and, in turn, the specimens’ remineralization behavior. For instance, Huang et al^28^ found that in pH-neutral environments, n-HA does not adequately remineralize subsurface lesions. This implies that if pH levels are too high, n-HA may lead to less stable enamel layers. Furthermore, the loss of minerals could not be considerably reduced by using n-HA alone on demineralized enamel surfaces.^29^ Because submicron enamel erosion defects or tiny holes cannot be penetrated by larger particles formed by the possible agglomeration of nHA.^28^ Furthermore, even though fluoride has been the most widely used remineralizing agent, a fluoride varnish alone is likely to be removed quickly due to the complicated oral environment, such as movement caused by the buccal muscle, tongue, mastication, saliva wash, and oral hygiene practices.^30^,^31^

Based on previous studies, the use of agarose hydrogel in a biomimetic mineralization model has shown promise for remineralizing human demineralized enamel.^32^ We hypothesized that the inclusion of EMD and n-HA could facilitate protein-mediated mineralization on apatite templates and overcome the drawbacks of using n-HA alone. Therefore, the current study aims to investigate the potential role of EMD-agarose in combination with n-HA as a novel composite hydrogel for remineralizing enamel to mimic the natural composition of tooth structure. The null hypothesis tested is that no significant difference exists between EMD-agarose hydrogel and n-HA-EMD-agarose hydrogel regarding enamel surface microhardness, mineral content (EDX spectroscopy), and surface topography (scanning electron microscope [SEM]) of the demineralized/remineralized dental enamel.

## Materials and Methods

### Ethical Consideration

This study was approved by the Research Ethics Committee, Faculty of Dentistry, Cairo University (approval no. 18766). Valid informed consent was obtained from all participants prior to collecting human teeth (impacted third molars) extracted for clinical reasons unrelated to this research.

### Specimen Preparation

A total of 27 impacted mandibular third molars with no detectable caries or abnormality were collected, ultrasonically cleaned to remove any tissue debris or deposits and rinsed with phosphate-buffered saline (PBS) (Capricorn-Scientific, Germany). Teeth were examined under a stereomicroscope (SMZ800N, Nikon, Japan), and any teeth with coronal surface abnormalities and cracks were excluded. All teeth were sectioned longitudinally in the mesio-distal direction, followed by horizontal sectioning to remove the roots with a diamond disk (Komet, Rock Hill, USA) under copious water coolant. Fifty-four buccal and lingual coronal tooth segments were obtained, each placed into custom-made plastic molds filled with self-curing acrylic resin (Acrostone Dental Factor, England) and cured for 24 h. Lastly, acid-resistant nail varnish was applied around the exposed enamel surface, leaving two equal window openings of approximately 3×3 mm (n = 108 windows).

### Demineralized Lesion Formation

Demineralized (erosive) lesions were created using a 37% phosphoric acid gel (Super Etch, SDI Limited, Australia) applied on the exposed enamel windows for one min, rinsed with deionized water for 60s, and thoroughly air-dried.^4^

### Phosphate Solution Preparation

A phosphate solution (0.26 M, pH 6.5) was prepared by dissolving sodium hydrogen phosphate (El-Gomhouria Co., Egypt) in deionized water, followed by the addition of sodium fluoride to achieve a final concentration of 500 ppm fluoride.^4^ This phosphate solution served as a mineral source for remineralization throughout the experiment.

### Hydrogels Preparation

Agarose hydrogel was prepared by dissolving 0.5 gm agarose powder (Vivantis, USA) in 100 mL of deionized water, maintaining a temperature of 55 °C until fully dissolved.^4^,^32^ The EMD-agarose hydrogel was prepared by adding 80 µg EMD (Straumann, Basel, Switzerland) to 400 µg calcium chloride (CaCl_2_) agarose hydrogel at 55 °C to achieve a final concentration of 1.5 mg/mL EMD.^4^ The n-HA-EMD-agarose hydrogel was prepared by incorporating n-HA powder (Nanostreams, Egypt) of particle size <20 nm into the prepared EMD-agarose hydrogel to reach a concentration of 10% n-HA, which was previously reported as optimum concentration for enamel remineralization.^33^ Following gelification, each specimen was placed in a sterile container filled with 20 mL of phosphate solution at 37 °C. The phosphate solution was replaced every 24 h, while the hydrogels were replaced every 48 h, with each specimen being ultrasonically cleaned with deionized water for 20s prior to replacement.^4^ Lastly, each coronal half randomly received an assigned hydrogel (either EMD-agarose or n-HA-EMD-agarose) of 2 mm thickness.

### Experimental Design Groups

**Figure 1:**
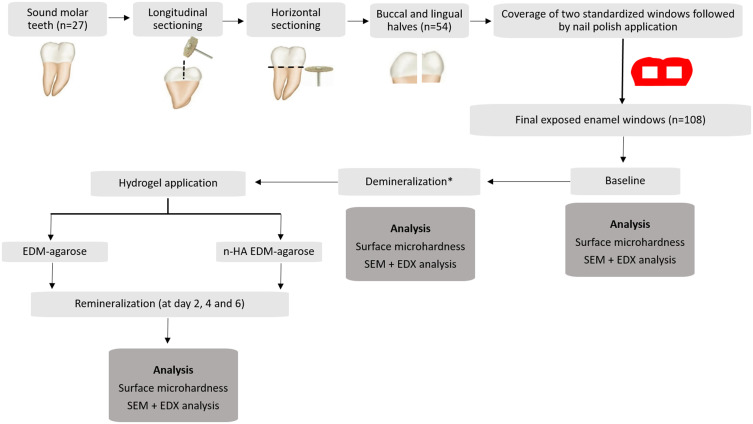
Flow diagram summarizing experimental steps. *Surface demineralization for all groups was performed with 37% phosphoric acid gel.

**Abbreviations**: EMD, enamel matrix derivative; n-HA, nano-hydroxyapatite; SEM, scanning electron microscope; EDX, energy dispersive x-ray spectroscopy.

Each coronal tooth half randomly received either the EMD-agarose or n-HA-EMD-agarose hydrogel, with each group further divided into three subgroups (n = 18/group) based on follow-up time (2, 4, and 6 days). Detailed experimental steps are illustrated in Figure 1.

### Assessment of the Remineralized Enamel

The exposed enamel windows for all groups—baseline (control), demineralized/erosive enamel, and hydrogel application—were assessed at day 2, 4, and 6, as previously reported.^4^ Six days was chosen as an end point to provide an indication about the initial effect on enamel remineralization. Each tooth segment contained two windows: one for surface microhardness analysis and the other for surface topography (scanning electron microscope; SEM) and mineral content (energy-dispersive x-ray spectroscopy; EDX) assessment. Detailed assessment steps are further described below.

### Surface Microhardness

Surface microhardness was measured using a Digital Display Vickers Microhardness Tester (Model HVS-50, Laizhou Huayin Testing Instrument Co., Ltd. China) connected with a Vickers diamond indenter and a 20x objective lens. Each specimen was subjected to a load of 200 g for 10s. Three indentations per specimen were recorded, and the average was calculated to represent the final microhardness. The indentations were distributed with a 100 µm distance between them, as previously prescribed.^34^,^35^ A built-in scaled microscope measured the length of the diagonal indentations, and Vickers values were converted into microhardness values.

Microhardness was calculated according to the following formula:

$$\mathrm{VHN=1.854\,P/d2,}$$

where VHN is Vicker hardness number (Kgf/mm^2^), P is the load (Kgf) and d is the length of the diagonals (mm).

### SEM and EDX Spectroscopy

Enamel samples were air-dried and mounted on the SEM (Quanta Field Emission Gun 250, FEI Company, Netherlands). All SEM images were obtained using a secondary electron live fiber detector under the magnification of 1000x – 5000x. Quantitative elemental analysis using EDX equipped with S- UTW detector (EDAX Inc., Mahwah, NJ, United States of America) was performed to assess and compare the chemical composition of calcium (Ca), phosphorous (P), and fluoride (F) weight percentage (wt. %). Each element was identified by its known wavelength on the *x*-axis, represented by a peak, and the peak intensity on the *y*-axis.

### Statistical Analysis

Based on previous studies,^36^,^37^ a total sample size of 54 (18/group) was determined sufficient to detect an effect size of approximately 4.0 with a power of 80%, and a significance level 5%. The sample size was calculated using G*Power program (University of Düsseldorf, Düsseldorf, Germany). Data were analyzed using the Statistical Package for the Social Sciences (SPSS; version 22 IBM Inc., Chicago, USA). Normality was confirmed, and two-way analysis of variance (ANOVA) followed by multiple comparison Tukey post hoc test when ANOVA yielded significant results, were employed. Repeated measures ANOVA compared groups at baseline, following demineralization, and after hydrogel application, with a p-value <0.05 considered statistically significant.

## Results

### Surface Microhardness

At baseline and after demineralization, no significant difference was detected between the microhardness of the EMD-agarose and n-HA-EMD-agarose hydrogels. However, following remineralization, a statistically significant higher mean microhardness was recorded in n-HA-EMD-agarose at all three time points (p = 0.027). The increase in exposure time of the applied EMD-agarose hydrogel showed a statistical significant increase in mean microhardness as recorded in day 6 (244.63 ± 2.76) as compared to both day 2 (187.76 ± 3.26) and day 4 (222.83 ± 5.57) (p < 0.05). Similarly, the n-HA-EMD-agarose hydrogel exhibited a statistical significant increase in mean microhardness in day 6 (260.87 ± 3.52) as compared to day 2 (211.43 ± 4.95) and day 4 (243.26 ± 3.17) (p < 0.05) (Table 1). In both groups, repeated measure ANOVA reported a significant increase in microhardness with time after remineralization, as compared to the samples at baseline and after demineralization (Supplementary Table 1).

**Table 1:** Descriptive Statistics and Comparison of Enamel Surface Microhardness Between Groups at Baseline, After Demineralization, and After Remineralization

	Material	Duration (days)	Mean±(SD)	Min	Max	Two-way ANOVA		
						Time	Material	Material×Time
**At Baseline**	EMD-agarose	2	252.5±(37.6)	201.2	312.1	0.957	0.902	0.983
		4	256.82±(17.76)	217.71	277.28			
		6	252.8±(45.3)	202.9	346.7			
	n-HA-EMD-agarose	2	254.86±(28.65)	224.02	315.51			
		4	255.73±(26.89)	210.54	291.74			
		6	254.57±(11.58)	244.60	280.98			
**After demineralization**	EMD-agarose	2	170.80±(20.30)	143.25	190.36	0.832	0.983	0.920
		4	177.30±(13.40)	159.99	199.99			
		6	178.53±(11.61)	158.43	196.02			
	n-HA-EMD-agarose	2	174.29±(28.52)	112.67	216.62			
		4	178.03±(23.06)	143.69	204.91			
		6	174.8±(47.1)	103.9	228.6			
**After remineralization**	EMD-agarose	2	187.76±(3.26)**^E^**	180.75	190.56	0.000*	0.000*	0.027*
		4	222.83±(5.57)**^C^**	210.37	229.14			
		6	244.63±(2.76)^B^	241.46	249.58			
	n-HA-EMD-agarose	2	211.43±(4.95)**^D^**	203.96	220.83			
		4	243.26±(3.17)**^B^**	238.02	249.14			
		6	260.87±(3.52)**^A^**	255.74	266.48			

**Notes**: *Statistically significant (significance level p < 0.05), Material×time; interaction between material and time, means with different superscript letters (E, C, B, D, A) indicate a significant difference in multiple comparison tests (Tukey’s post-hoc test).

**Abbreviations**: EMD, enamel matrix derivative; n-HA, nano-hydroxyapatite; SD, standard deviation.

### EDX Spectroscopy

#### Calcium (Ca) Elemental Analysis

**Figure 2:**
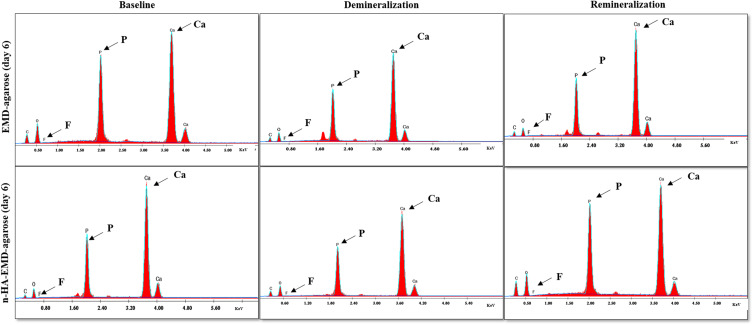
Representative charts of EDX spectroscopy elemental composition of calcium (Ca), phosphorus (P), and fluoride (F) weight percentage of EMD-agarose and n-HA-EMD-agarose hydrogels at day 6 (Ca, P and F peaks are indicated by arrows). Overall, higher peaks of Ca, P and F are seen in both hydrogels following remineralization at day 6.

**Figure 3:**
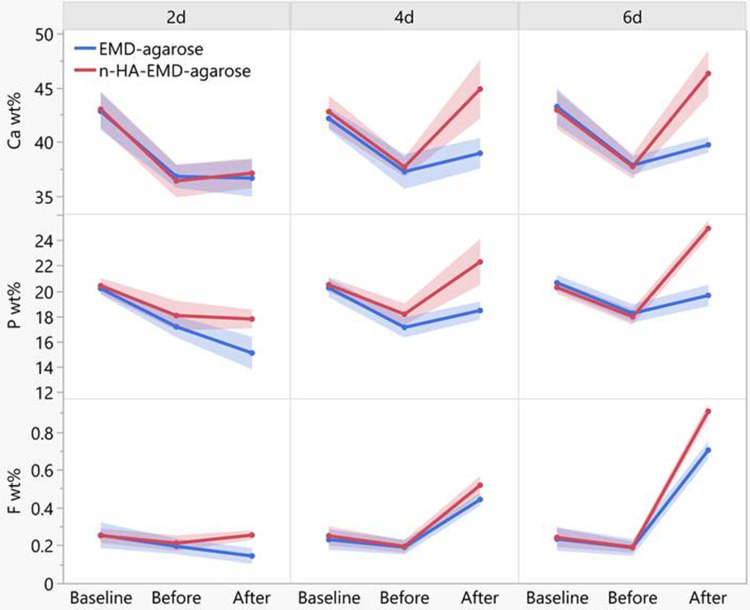
Line chart representing the calcium (Ca), phosphorous (P) and fluoride (F) weight percentage (wt.%) for different tested groups. A significant increase in Ca, P and F content following application of n-HA-EMD-agarose hydrogel at day 6 was evident. The full data can be accessed in the Supplementary Tables file.

Quantitative elemental analysis of Ca wt.% revealed no significant difference in both hydrogel groups at baseline or after demineralization. However, following remineralization, the n-HA-EMD-agarose hydrogel showed a statistically significant higher mean Ca wt.% compared to EMD-agarose at 4 and 6 days (p < 0.05). The combined n-HA-EMD-agarose hydrogel also resulted in a statistically significant increase in mean Ca wt.% at day 6 (46.3 ± 2.78) and day 4 (44.88 ± 3.55) as compared to day 2 (37.09 ± 1.79) (p < 0.05) (Figures 2 and 3). Repeated measure two-way ANOVA indicated a significant increase in Ca wt.% with time and following remineralization in the n-HA-EMD-agarose group (p < 0.05), while a non-significant difference was seen in EMD-agarose hydrogel (Supplementary Tables 1 and 2).

#### Phosphorous (P) Elemental Analysis

Similar to Ca, no significant difference was found in P wt. % between the two hydrogel groups at baseline and after demineralization. After remineralization, the n-HA-EMD-agarose hydrogel exhibited significantly higher P wt. % compared to EMD-agarose at all time points (p = 0.028) (Figures 2 and 3). The EMD-agarose hydrogel reported a statistically significant higher mean values in day 6 (19.64 ± 1.092) as compared to day 2 (15.1 ± 1.673), and at day 4 (18.45 ± 0.923) in comparison to day 2 (15.1 ± 1.673) (p < 0.05). A similar pattern was noted for n-HA-EMD-agarose in which statistically significant higher mean P values were recorded at day 6 (24.92 ± 0.826) as compared to both day 2 (17.78 ± 0.961) and day 4 (22.29 ± 2.352) (p < 0.05). Repeated measure two-way ANOVA indicated a significant increase in P wt. % with time and following remineralization in both hydrogels (Supplementary Tables 1 and 3).

#### Fluoride (F) Elemental Analysis

Quantitative elemental analysis of F wt. % revealed no significant difference between both EMD-agarose and n-HA-EMD-agarose at baseline or after demineralization. Following remineralization, a statistically significant higher mean F wt. % was recorded in n-HA-EMD-agarose as compared to EMD-agarose in all time points (p = 0.002) (Figures 2 and 3). A statistically significant higher mean F value was recorded in EMD-agarose at day 6 (0.7033 ± 0.0624) as compared to day 2 (0.1433 ± 0.0543) and day 4 (0.4422 ± 0.0429) (p < 0.05). In a similar pattern, a statistically significant higher mean F value was recorded in n-HA-EMD-agarose at day 6 (0.909 ± 0.053) as compared to both day 2 (0.254 ± 0.033) and day 4 (0.518 ± 0.064) (p < 0.05). Repeated measure two-way ANOVA indicated a significant increase in F wt.% with time and after remineralization in both hydrogels (EMD-agarose and n-HA-EMD-agarose) (Supplementary Tables 1 and 4).

#### SEM Analysis

**Figure 4:**
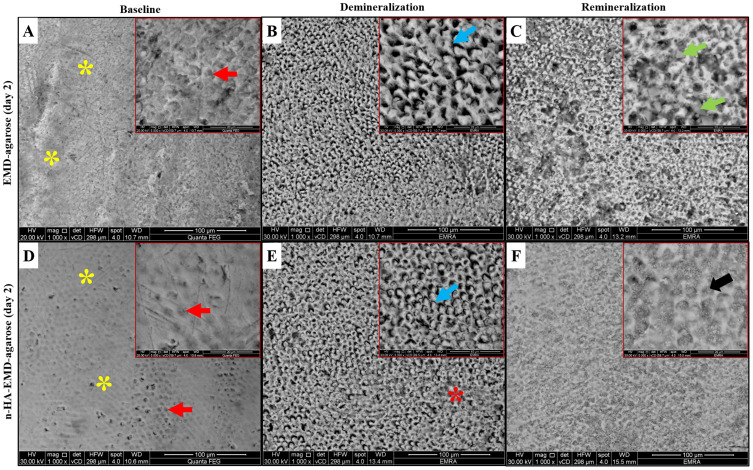
Representative SEM images of EMD-agarose and n-HA-EMD-agarose at day 2, showing areas of aprismatic enamel (yellow asterisks) and enamel rod ends (red arrows) at the baseline. After demineralization, areas of type II (blue arrows) and type III (red asterisks) etching patterns were seen. Following remineralization, EMD-agarose showed obliteration of rod cores by mineral deposits (green arrows), while in n-HA-EMD-agarose the outlines of the rods were still visible despite generalized obliteration of rod cores (black arrow). (**A** and **D**) at baseline; (**B** and **E**) after demineralization; (**C** and **F**) at day 2 remineralization.

**Figure 5:**
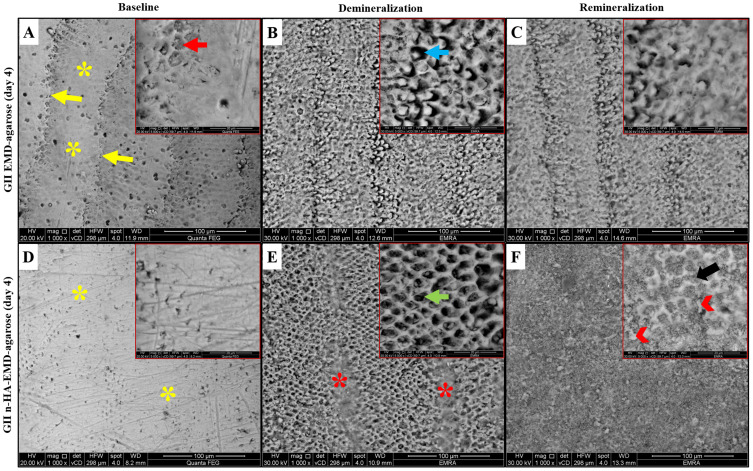
Representative SEM images of EMD-agarose and n-HA-EMD-agarose at day 4, showing areas of aprismatic enamel (yellow asterisks), enamel rod ends (red arrows) and clearly defined perikymata grooves (yellow arrows) at the baseline. After demineralization, areas of type I (green arrow), type II (blue arrow) and type III (red asterisks) etching patterns were evident. Following remineralization, EMD-agarose revealed a relatively smooth enamel surface in which enamel rods became less visible. The deposition of n-HA on the surface and obliteration of the rod cores (black arrow) were seen in the n-HA-EMD-agarose group with the formation of micro-clusters on the surface (arrow heads). (**A** and **D**) at baseline; (**B** and **E**) after demineralization; (**C** and **F**) at day 4 remineralization.

**Figure 6:**
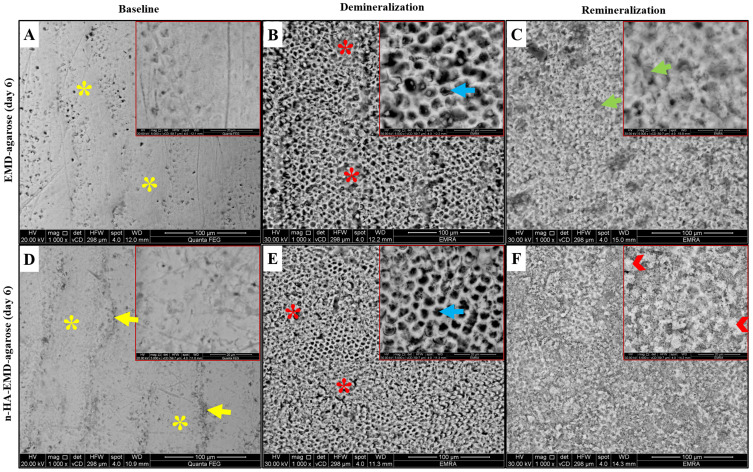
Representative SEM images of EMD-agarose and n-HA-EMD-agarose at day 6, showing areas of aprismatic enamel (yellow asterisks) and clearly defined perikymata grooves (yellow arrows) at the baseline. After demineralization, areas of type II (blue arrows) and type III (red asterisks) etching patterns were seen. Following remineralization, EMD-agarose showed generalized obliteration of rod cores resulting in a relatively homogenous surface (green arrows), while complete disappearance of porous inter-rod and rod enamel structures by a thick apatite layer, with exception to a few small areas, were seen in the n-HA-EMD-agarose hydrogel group (arrow heads). (**A** and **D**) at baseline; (**B** and **E**) after demineralization; (**C** and **F**) at day 6 remineralization.

Representative SEM images are shown in Figures 4–6. At baseline, the enamel surface revealed complete or partial loss of aprismatic enamel with enamel rod ends appearing with varying depths. Various enamel surface structures were visible such as; a relatively smooth surface with a uniform aprismatic surface layer, or apparent perikymata grooves. Following demineralization, various etching patterns were seen across all groups including, type I etching pattern in which a distinct hollowing of rod centers with relatively intact rods’ peripheries exists. Rod ends also showed type II etching pattern with preferential loss of inter-prismatic substance and areas of type III etching pattern without a distinct rod morphological appearance were also seen.

Following remineralization, the EMD-agarose revealed various changes with time starting with obliteration of rod cores by mineral deposits (day 2; Figure 4C), followed by a smooth enamel surface with less visible enamel rods due to mineral deposition at day 4 (Figure 5). At day 6, EMD-agarose denoted a generalized obliteration of rod cores resulting in a smooth and homogenous surface with slightly visible rods’ outlines (Figure 6). In regards n-HA-EMD-agarose hydrogel, surface remineralization changes at day 2 revealed a generalized obliteration of rod cores due to n-HA deposition (Figure 4). At day 4, n-HA-EMD-agarose revealed an increased deposition of n-HA on the surface obliterating the rod cores. Upon magnification, it was seen that the nucleating n-HA gradually began to grow and form micro-clusters on the enamel surface (Figure 5). At day 6, in most areas a complete disappearance of porous inter-rod and rod enamel structures by a thick apatite layer was evident. Higher magnification interpretation revealed the n-HA micro-clusters were gradually enlarging on the enamel surface obscuring the rods’ outline (Figure 6). Overall, the n-HA-EMD-agarose hydrogel demonstrated a superior potential to obliterate enamel rods when compared to EMD-agarose hydrogel.

## Discussion

The adoption of regenerative-based approaches in the dental field is of continuous significant interest.^21^ Specifically, the biomimetic mineralization and development of enamel-like apatite structures is regarded as an attractive alternative restorative option.^7^,^23^ The biomimetic mineralization mechanism is a non-classical crystallization method mediated by organic matrix particles.^38^ The organic matrix functions as a template in the biomineralization process and regulates the mineral crystallites through a molecular interaction between the polymer and minerals with a sequestering mechanism.^38^ At the organic surface, the ion or cluster forms amorphous initial particles that later accumulate to form orientated crystallization.^39^,^40^

Therefore, it is of clinical significance to explore valid strategies to optimize enamel remineralization and re-establish its normal function. Various hydrogels, such as agarose-based and EMD-based hydrogels, have been tested.^4^,^11^,^32^ Agarose helps form agarose fiber-nanoscale-amorphous calcium phosphate complex precursors when combined with calcium and phosphate ions.^4^,^13^,^41^ These complexes stay consistent in size due to the gel’s structure,^15^,^16^ and eventually grow into a dense layer like tooth enamel.^13^ To the authors’ knowledge, the combined addition of n-HA to EMD-agarose hydrogels has not been previously tested. To address this knowledge gap in the literature, this study aimed to investigate and compare the possible biomimetic effect of an agarose-based hydrogel loaded with EMD alone or combined with n-HA in remineralizing human demineralized enamel. Since amelogenesis is a prolonged process, where the dense layer of enamel crystals forms at a very slow rate of few µm per week, therefore enamel remineralization process requires the repeated application of the remineralizing material to achieve sufficient enamel hardness.^42^ Enamel remineralization involves complex interactions between the enamel surface and the remineralizing agents. Therefore, based on the previous literature,^4^,^11^ the application times in the present study were at 2, 4, and 6 days. Six days was chosen as an end point and aimed to provide an initial indication of the effect of the applied hydrogel on enamel remineralization.

Previous literature has shown that the agarose-based hydrogel microenvironment is a suitable model for the hierarchical formation and growth of tooth-like crystals.^12^,^32^ Therefore, in this study, an agarose-based hydrogel was selected to mimic dental enamel’s gel-like organic matrix environment. The agarose-based hydrogel also acts as a reservoir of minerals and a dynamic interface to transport the minerals to the enamel surface for mineral transformation. This diffusion pathway of minerals into the hydrogel results in oriented fluoridated HA crystallization.^4^ Additionally, the ability of n-HA to penetrate the surface porosities and act as a building scaffold is of clinical significance.^43^ The n-HA ability to absorb Ca and P ions from the remineralizing solution and deposit the absorbed ions onto the superficial enamel surface is an additional clinical advantage. The reported mechanism is by filling the spaces between the enamel calcium crystals, thus resulting in a uniformly crystalline enamel structure.^43^ According to the previous literature, the inherent nature of n-HA, such as size of calcium phosphate crystals, chemical composition, and structural resemblance to enamel apatite, are regarded as crucial factors in their mode of action and remineralization process.^28^ The hybrid addition of n-HA to an agarose-based hydrogel was of research focus in the current study.

Overall, results of the current study rejected the null hypothesis as the combined addition of n-HA to the EMD-agarose hydrogel resulted in improved enamel surface microhardness and higher mineral content as compared to only EMD-agarose hydrogel. Additionally, a significant increase in microhardness values with time following remineralization was recorded, in which surface microhardness values showed a pronounced recovery at day 6 (260.87 ± 3.52) with values close to the baseline recording (254.57 ± 11.58).

However, it must be mentioned that the increase in surface hardness following remineralization stage might not recover to the initial hardness values before demineralization. This could be explained due to the loosely packed newly formed crystals on the surface layer as compared to the densely packed crystals of the natural enamel.^11^ This was further supported by the findings of Han et al who demonstrated that mineral uptake was significantly higher in teeth treated with an amelogenin-derived peptide in an animal carious tooth model.^41^ They also hypothesized that amelogenin functions via surface binding and spontaneous self-assembling into fibrillar scaffolds in response to particular environmental stimuli, creating a biomimetic scaffold capable of nucleating the synthesis of hydroxyapatite.^41^

Furthermore, analysis of the enamel surface topography of the n-HA-EMD-agarose hydrogel under the SEM revealed the deposition of n-HA inside the pores (created after demineralization). These results are in-line with Swarup & Rao, who reported the deposition of a homogeneous thick apatite layer covering the porous demineralized enamel surface.^44^ The n-HA deposition observed in the present work can be explained by the capacity of n-HA to biomimetically heal damaged enamel by depositing on natural tissue or, conceivably, by filling up microspores and defects in demineralized teeth.^45^

In the current study, SEM results of EMD-agarose at different follow-up times were in accordance with previous literature.^11^ Cao et al demonstrated that the regenerated crystals in the presence of EMD were more dense, thick, and orderly packed in comparison to those formed without EMD in the agarose hydrogel model.^11^ On the other hand, they revealed that the degree of structural perfection in the newly regenerated enamel prism-like tissue without EMD was higher than that with EMD and further attributed crystal imperfections due to rapid crystal growth.^11^

Mineral content elemental analysis using EDX indicated a decrease in Ca, P, and F wt. % following demineralization of enamel surface in all groups. These results are in-line with the microhardness results, in which a significant decrease in the microhardness values following demineralization was apparent. However, following remineralization, results of this study reported a time-dependent positive effect of n-HA on enamel remineralization as reflected by increase in Ca, P, and F wt. %. Indeed, the clinical application of n-HA has been associated with a significant improvement in enamel surface microhardness and successful remineralization of early caries lesions.^28^,^33^ This precipitation process could be translated into faster remineralization process and the formation of fluorapatite of higher hardness properties in comparison to hydroxyapatite.^46^

The authors acknowledge study limitations such as the in-vitro design and the short-term assessment duration. Furthermore, characterization of the prepared hydrogel, assessing various concentration of n-HA, the effect of the hydrogels on bonding performance, stability and color re-establishment of the demineralized enamel needs to be further assessed. However, the strengths of this study lie within the novel application of n-HA EDM-agarose hydrogel for the biomimetic regeneration of early demineralized enamel lesions. Future studies should also focus on the identification of hydroxyapatite crystals, such as X-ray Diffraction (XRD) analysis, and to assess the thickness of the remineralized enamel as a key indicator to rate a materials clinical value and effectiveness in enamel repair. Study designs such as finite element analysis to assess the biomechanical behaiour of new hydogels in a controlled standardized setting would be advantageous.^47^ In addition to the reported improved surface microhardness and essential mineral deposition, this novel hydrogel model possesses advantages, including non-complex preparation steps and relatively low production cost, which can be transferred from bench-side to chair-side. Further, well-controlled, randomized clinical studies are required to draw sound conclusions.

## Conclusions

In conclusion, the combined n-HA-EMD-agarose hydrogel demonstrated enhanced mineral content deposition and improved surface microhardness of remineralized enamel, indicating its potential as a biomimetic approach for dental enamel repair. The synergistic effect of n-HA and EMD in the agarose hydrogel matrix promotes the remineralization of demineralized enamel, closely mimicking the natural composition and structure of tooth enamel. Further *in-vivo* studies and clinical trials are warranted to validate the effectiveness and long-term stability of this novel biomimetic hydrogel in real-world settings.

## Data Availability

Dataset used and analyzed data can be available from corresponding author on reasonable request.
